# Phenolic Characterisation of *Citrus* Peel Aqueous Extract: By-Products of Hydrodistillation and the Effect of Enzymatic Pretreatment

**DOI:** 10.3390/plants15182871

**Published:** 2026-09-19

**Authors:** Antonela Ninčević Grassino, Marija Penić, Sandra Pedisić, Sven Karlović, Maja Dent

**Affiliations:** Faculty of Food Technology and Biotechnology, University of Zagreb, Pierottijeva 6, 10000 Zagreb, Croatia; doktoricamare@gmail.com (M.P.); spedisic@pbf.hr (S.P.); skarlovi@pbf.hr (S.K.)

**Keywords:** aqueous extracts, citrus peel, enzymatic pretreatment, hydrodistillation, phenolic compounds, UPLC–MS/MS

## Abstract

This study aimed to characterize the phenolic composition of aqueous extracts obtained as by-products of hydrodistillation of orange, mandarin, and clementine peels and to evaluate the effect of enzymatic pretreatment on phenolic recovery. Different enzymatic pretreatments were applied prior to hydrodistillation, and the resulting extracts were analyzed by UPLC-MS/MS. Differences in the mass fractions of phenolic compounds were observed among pretreatments, with the magnitude of these changes depending on the citrus species and extraction conditions. Quercetin and kaempferol were the predominant phenolic compounds in all three citrus species. The highest sum of the mass fractions of quantified phenolic compounds was observed for mandarin peel following combined enzyme pretreatment in citrate buffer (19.73 µg/g) and for clementine peel following xylanase pretreatment in water (34.94 µg/g). For orange peel, the highest value was obtained following combined enzyme pretreatment in citrate buffer (10.88 µg/g), although no increase in the sum of the mass fractions of quantified phenolic compounds was observed compared with the corresponding control. Overall, the observed changes following enzymatic pretreatment were species-dependent, with the most pronounced increases in phenolic mass fractions found for mandarin and clementine peels.

## 1. Introduction

*Citrus* fruits, belonging to the *Rutaceae* family, are among the most extensively cultivated agricultural crops globally. According to the Food and Agriculture Organization of the United Nations (FAO), total global production is projected to reach approximately 174 million metric tons in 2026, spanning a cultivation area of 10.2 million hectares [1]. The most commercially significant citrus species include sweet and sour orange (*Citrus* × *sinensis*, *Citrus* × *aurantium*), mandarin (*Citrus reticulata*), grapefruit (*Citrus* × *paradisi*), pomelo (*Citrus maxima*), lemon (*Citrus* × *limon*), citron (*Citrus medica*), lime (*Citrus* × *aurantiifolia*), kumquat (*Citrus japonica*), and their respective hybrids [2,3], which are predominantly consumed either as fresh fruit or processed juices. Approximately one-third of global citrus production is industrially processed, generating by-products that account for 50–60% of the fresh fruit mass and mainly include peels, pulp, pomace, and seeds [4]. Although these materials have traditionally been used as animal feed [5,6], increasing attention has been directed towards their valorization as sources of functional and bioactive compounds for applications in the food, pharmaceutical, and cosmetic industries, as well as in the development of biodegradable packaging and edible films [2,3,7,8,9,10]. Their expanded utilization is primarily attributed to the fact that citrus by-products are rich sources of vitamins, pectin, essential oils, alkaloids, limonoids, and flavonoids, which act as bioactive compounds closely linked to their functional properties [5,11,12,13,14,15]. The polyphenolic fraction is predominantly characterized by flavonoids, stilbenes, lignans, and tannins, alongside phenolic acids such as caffeic, cinnamic, *p*-coumaric, ferulic, and gallic acids [16,17,18,19]. Flavanones—specifically hesperidin and naringenin as the major components—occur in addition to neohesperidin, naringin, narirutin, eriocitrin, apigenin, quercetin, catechin, and rutin as the primary polyphenols in citrus by-products [17,18,19,20,21,22,23,24].

Although the phenolic composition of citrus peels has been extensively investigated using various extraction techniques, including conventional extraction [25,26,27], solid–liquid extraction [28], ultrasound-assisted extraction [25,28,29,30,31], microwave-assisted extraction [32,33], cold plasma [24], and pressurized extraction [34], studies on aqueous extracts obtained after hydrodistillation remain scarce. The composition and concentration of phenolic compounds vary considerably among citrus species and anatomical parts, with higher levels generally reported in peels and other solid fractions. Previous studies have mainly focused on lemon, orange, mandarin, and grapefruit peels, whereas clementine peel remains comparatively unexplored. Gómez-Mejía et al. [27] reported the phenolic composition of clementine peel based on ethanolic extracts, while Gómez-Mejía et al. [35] characterized aqueous extracts from orange and mandarin peels after hydrodistillation without enzymatic pretreatment. Enzyme-assisted extraction has been investigated to improve the recovery of phenolic compounds from citrus by-products. Enzymatic treatment has been shown to enhance phenolic release and recovery, particularly when carbohydrases such as pectinase, Viscozyme L, and Pectinex Ultra SP-L are applied to citrus by-products [36,37,38]. However, these studies focused on direct enzymatic extraction rather than enzymatic pretreatment prior to hydrodistillation. Thus, the effect of enzymatic pretreatment on the phenolic composition of aqueous residues generated during hydrodistillation remains insufficiently characterized. Hydrodistillation is widely used for essential oil isolation and also generates aqueous fractions containing water-soluble non-volatile compounds released during boiling. In our previous studies [39,40], hydrodistillation was applied to clementine, mandarin, and orange peels to evaluate volatile compounds in essential oils and hydrolates, while the residual aqueous phase was not investigated. Therefore, the objective of this study was to evaluate the effect of enzymatic pretreatment with cellulases, xylanases, pectinases, and their combinations prior to hydrodistillation on the phenolic composition of aqueous extracts from orange, mandarin, and clementine peels, with particular emphasis on individual phenolic compounds and the sum of the mass fractions of quantified phenolic compounds.

## 2. Results and Discussion

### 2.1. Phenolic Composition of Orange, Mandarin and Clementine Peel Aqueous Extracts

**Figure 1:**
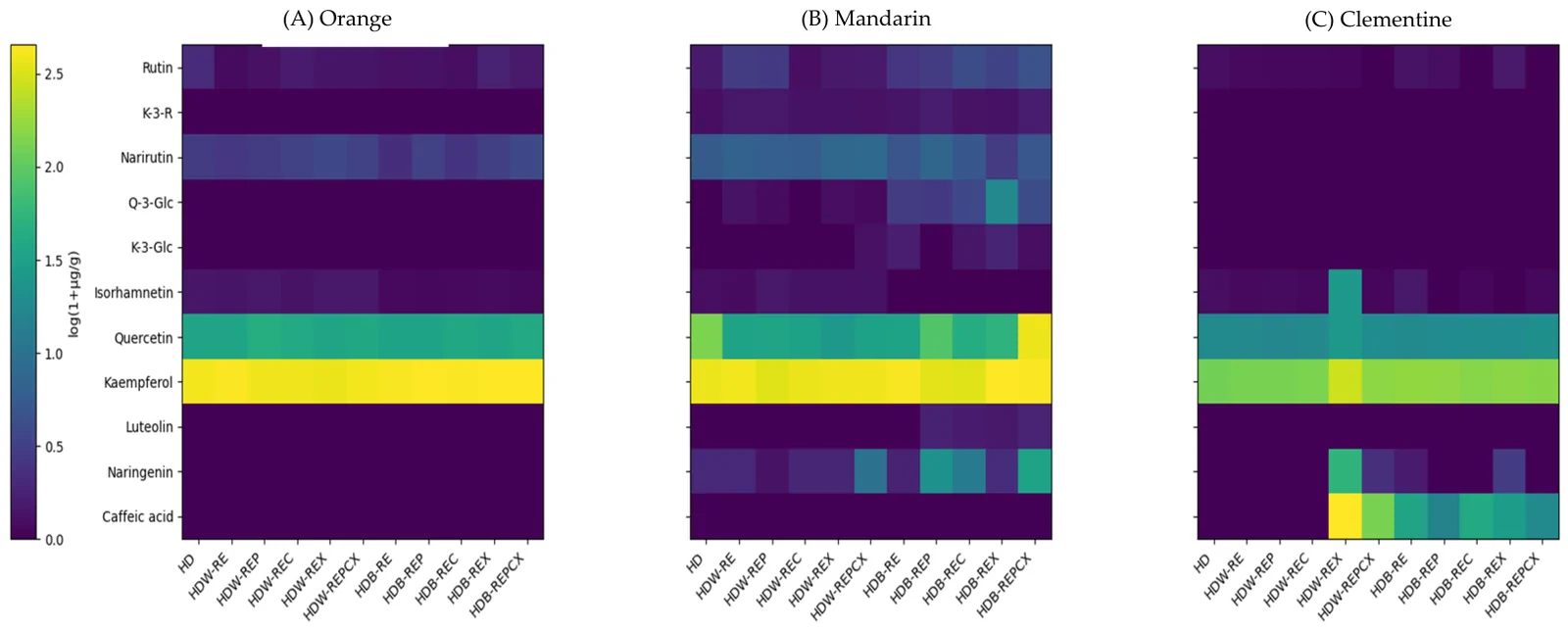
Log-transformed heatmaps of phenolic composition in citrus peel water extracts: (**A**) orange, (**B**) mandarin, and (**C**) clementine, obtained under different pretreatment conditions. HD—hydrodistillation without pretreatment (no—pretreatment control); HDW—hydrodistillation with water (no—enzyme control); HDB—hydrodistillation with buffer (no-enzyme control); RE—reflux extraction without enzyme; REP—reflux extraction with pectinase-assisted pretreatment; REC—reflux extraction with cellulase-assisted pretreatment; REX—reflux extraction with xylanase-assisted pretreatment; REPCX—reflux extraction with combined enzyme pretreatment (pectinase/cellulase/xylanase). Phenolic compounds: K-3-R, kaempferol-3-O-rutinoside; Q-3-Glc, quercetin-3-O-glucoside; and K-3-Glc, kaempferol-3-O-glucoside.

The phenolic composition of aqueous extracts obtained from citrus peels after hydrodistillation has been scarcely investigated. The only available study, reported by Gomez Mejia et al. [35], characterized the phenolic profile of aqueous extracts from orange and mandarin peels without the use of enzymatic pre-treatments. Nevertheless, the phenolic composition of citrus peels has been extensively investigated using various extraction techniques, including conventional extraction [25,26,27], solid–liquid extraction [28], ultrasound-assisted extraction [25,29,30,31], microwave-assisted extraction [31,32] or pressurized extraction [33]. These studies have been primarily focused on lemon, orange, mandarin, and grapefruit peels. In contrast, clementine peel remains largely unexplored. To date, only Gómez-Mejía et al. [27] have reported the phenolic composition of clementine peel, based on the analysis of ethanolic extracts. In citrus juice production, enzymatic extraction is used to treat by-products consisting of peels, seeds, and pulp in order to enhance the isolation and conversion of bioactive phenolic compounds [36]. It has been shown that enzyme-assisted extraction, particularly using carbohydrases such as pectinase and Viscozyme L on orange peel, as well as Pectinex Ultra SP—L on pomelo by-products, can significantly enhance the recovery of phenolic compounds [37,38]. This research gap underscores the limited understanding of enzymatic pretreatment prior to hydrodistillation, particularly its mechanisms and efficacy in enhancing aqueous extraction processes. During hydrodistillation for essential oil isolation, considerable amounts of aqueous extract rich in bioactive compounds are generated [34]. However, this aqueous fraction remains largely underutilized despite representing a valuable source of phytochemicals. Its utilization could enable the simultaneous recovery of bioactive compounds alongside essential oils, contributing to a more sustainable and resource-efficient biorefinery approach while reducing the need for organic solvents and additional extraction processes, including conventional as well as ultrasound- and microwave-assisted extraction methods. In this study, aqueous extracts obtained as by-products of Clevenger hydrodistillation during the isolation of essential oils from enzymatically pretreated orange, mandarin, and clementine peels were analyzed using UPLC-MS/MS (Table 1, Table 2 and Table 3, Supplementary Figure S1). To compare phenolic profiles among different pretreatments, a heat map with hierarchical clustering was generated based on UPLC-MS/MS data (Figure 1A–C). The obtained results revealed distinct phenolic profiles among aqueous extracts obtained from orange, mandarin, and clementine peels after hydrodistillation, as illustrated by the UPLC-MS/MS heatmap (Figure 1A–C). Regardless of the enzymatic pre-treatment applied, kaempferol and quercetin were the predominant phenolic compounds in all citrus species. In addition, orange and mandarin extracts contained high levels of narirutin, whereas this flavanone glycoside was virtually absent in clementine extracts. Mandarin extracts exhibited the greatest diversity of phenolic compounds, including relatively higher levels of quercetin-3-glucoside, naringenin, and rutin compared with orange and clementine. In contrast, clementine extracts showed a simpler phenolic profile, dominated by kaempferol and quercetin, with caffeic acid detected only in selected enzyme-treated samples. Overall, species-related differences appeared more pronounced than differences associated with enzymatic pre-treatment. Despite these interspecies variations, quercetin and kaempferol were consistently identified in the aqueous extracts of all three citrus species. Quercetin was detected at concentrations of 2.78, 7.10, and 3.11 µg/g, whereas kaempferol reached 7.46, 7.58, and 10.70 µg/g in orange, mandarin, and clementine extracts, respectively, indicating that these flavonols are characteristic constituents of citrus peel aqueous extracts. M’Hiri et al. [41] reported that quercetin and kaempferol are among the most abundant flavonols in citrus peels, predominantly occurring in glycosidic forms. Additionally, flavonones such as narirutin (1.09 µg/g) and naringenin (2.46 µg/g) were detected in mandarin peel extracts. Clementine peel extracts exhibited comparatively higher levels of caffeic acid (13.25 µg/g), kaempferol (10.70 µg/g), naringenin (4.64 µg/g), isorhamnetin (3.19 µg/g), and quercetin (3.11 µg/g). In addition, numerous studies have confirmed the presence of narirutin, naringenin, and hesperidin as major flavonoids in peels of various citrus species. Gomez-Mejia [27] reported high-yield hesperidin (673 mg/g) in clementine peel ethanolic extract. Safdar et al. [25] identified hesperidin (92.94 μg/g), quercetin (29.78 μg/g), and kaempferol (16.85 μg/g) as the major flavonoids in mandarin peel following ultrasound-assisted extraction using methanol and ethanol, while ferulic acid was the predominant phenolic acid (102.13 μg/g). Likewise, Morales et al. [31] reported very high yields of hesperidin (9445 μg/g) and narirutin (1719 μg/g) following ultrasound-assisted extraction of mandarin peel with aqueous ethanol. When water was used as the extraction solvent, substantially lower yields of these compounds (hesperidin 3275 μg/g, and narirutin 1263 μg/g) were obtained, highlighting the important role of solvent selection in phenolic recovery. Similarly, Hayat et al. [32] reported naringenin and kaempferol contents (24.1–29.3 μg/g and 157.1–203.5 μg/g, respectively) after microwave-assisted extraction of mandarin peel. Zapata et al. [28] determined narirutin and quercetin yields (3600 μg/g and 73 μg/g, respectively) in methanolic orange peel extracts, while Cebadera-Miranda et al. [26] reported concentrations of up to 930 μg/g narirutin and 420 μg/g quercetin in homogenized fresh blood orange pulp. Gómez-Mejía et al. [27] reported naringin concentrations of 4.32 and 1.72 mg/g extract in orange and clementine peel ethanolic extracts, respectively. Quercetin was detected only in orange peel extracts and at very low levels. To the best of our knowledge, the only study that has investigated the phenolic composition of aqueous extracts obtained after hydrodistillation of citrus peels is that of Gómez-Mejía et al. [35]. Their results revealed marked species-dependent differences in total phenolic content, with aqueous lemon peel extracts containing approximately 22 mg of total phenolics per gram of extract, whereas orange and mandarin peel extracts contained approximately 5 mg/g extract. Regarding individual phenolic compounds, glycosides of diosmetin, luteolin, and eriodictyol were identified as the predominant phenolics in orange peel aqueous extracts, while luteolin-C-hexoside was the major phenolic constituent in mandarin peel extracts. In contrast, glycosylated derivatives of naringenin and quercetin were detected in orange peel extracts at considerably lower concentrations, generally below 1 mg/g extract. Direct comparison of these results with those obtained in the present study is not possible because the phenolic contents reported by Gómez-Mejía et al. [35] were expressed on an extract basis (mg/g extract), whereas the results of the present study were expressed on a peel basis. Furthermore, their study did not include enzymatic pre-treatment prior to hydrodistillation. Nevertheless, the available evidence demonstrates that aqueous extracts generated as by-products of hydrodistillation contain appreciable amounts of phenolic compounds, whose qualitative and quantitative composition is strongly influenced by citrus species. In addition, differences in cultivation conditions, geographical origin, maturity stage, and other agronomic factors may further contribute to the variability observed among citrus peel extracts. A review of the available literature confirms that citrus peels are rich sources of phenolic compounds with considerable bioactive potential. The major compounds identified in the present study, namely quercetin, kaempferol, naringenin, and caffeic acid, are consistent with phenolic profiles previously reported for citrus peel extracts. Although the concentrations of individual phenolic compounds were generally lower than those reported for ultrasound-assisted, microwave-assisted, and solvent-based extraction methods, such differences are expected due to variations in extraction conditions, solvents, plant materials, and analytical approaches. While Gómez-Mejía et al. [35] demonstrated that aqueous extracts obtained as by-products of hydrodistillation are a valuable source of phenolic compounds, studies employing conventional solvent extraction, ultrasound-assisted extraction, and microwave-assisted extraction [25,26,28,31,32] further confirm the richness of citrus peels in bioactive phenolics. Collectively, these findings highlight the potential for valorization of hydrodistillation by-products as value-added ingredients within sustainable biorefinery strategies and circular bioeconomy frameworks.

### 2.2. Effect of Enzymatic Pre-Treatments in Modulating the Phenolic Composition of Orange, Mandarin and Clementine Peel Aqueous Extracts

The effect of enzymatic pretreatment prior to hydrodistillation on the phenolic composition of aqueous extracts obtained from orange, mandarin, and clementine peels was evaluated within each citrus species (Table 1, Table 2 and Table 3). Considering that the replicate measurements represented repeated analytical determinations of the same prepared sample rather than independent experimental replicates, comparisons among pretreatments were interpreted primarily descriptively, based on the observed mass fractions and trends. Given the limited number of studies investigating enzymatic pretreatment before hydrodistillation and its influence on the phenolic profile of the resulting aqueous extracts, the obtained results were compared with the available literature on enzyme-assisted extraction of citrus by-products. Previous studies have demonstrated that enzymatic treatments enhance the recovery of phenolic compounds and modify the phenolic composition of citrus peel extracts by promoting the degradation of structural polysaccharides [36,37,38]. In the present study, the sum of the mass fractions of quantified phenolic compounds of aqueous citrus peel extracts varied according to the pretreatment applied. Enzymatic pretreatment increased the sum of the mass fractions of quantified phenolic compounds in mandarin peel from 13.22 to 19.73 µg/g and in clementine peel from 9.89 to 34.94 µg/g. In contrast, no increase in the sum of the mass fractions of quantified phenolic compounds was observed for orange peel, for which the maximum value reached 10.88 µg/g. These results indicate that the effect of enzymatic pretreatment on phenolic compound recovery was species-dependent, with a more pronounced enhancement observed for mandarin and clementine peels, whereas no clear improvement was observed for orange peel. Therefore, enzyme-assisted processing may have potential for the valorization of citrus by-products, although its effectiveness appears to depend on the citrus species and should not be generalized across all citrus peels. The observed changes in phenolic mass fractions varied depending on the citrus matrix and extraction medium, with more pronounced increases observed for mandarin and clementine peels. In orange and mandarin peels, the effect of xylanase was pronounced not only in aqueous systems but also under buffered conditions. In both media, xylanase alone and combined enzyme systems containing xylanase and auxiliary enzymes were associated with higher mass fractions of several phenolic compounds. Furthermore, synergistic enzyme mixtures in buffered systems were associated with higher overall mass fractions of quantified phenolic compounds in mandarin peel, while in orange peel they promoted the recovery of certain individual flavonoids without increasing the sum of the mass fractions of quantified phenolic compounds. In contrast, clementine peel showed a more pronounced response primarily to xylanase treatment in water, indicating species-specific differences in cell wall composition and the accessibility of its components to enzymatic action. Overall, xylanase and combined enzyme systems were associated with higher observed mass fractions of phenolic compounds, particularly in mandarin and clementine peels, whereas the response of orange peel was more limited and did not result in an increase in the sum of the mass fractions of quantified phenolic compounds. These observations are consistent with the proposed role of the synergistic degradation of pectin, cellulose, and hemicellulose in facilitating the release of phenolic compounds from citrus processing by-products and suggest the potential of enzyme-assisted pretreatment prior to hydrodistillation as an effective strategy for improving the recovery of valuable bioactive compounds. These findings are consistent with previous studies demonstrating that optimized enzymatic treatments promote the degradation of structural polysaccharides and facilitate the release of bound phenolic compounds [42,43,44]. The enhanced recovery observed in the present study may be attributed to the complementary action of pectinase, cellulase, and xylanase, which hydrolyze pectin, cellulose, and hemicellulose, thereby disrupting cell wall integrity, increasing cell permeability, and facilitating the release of phenolic compounds entrapped within the cellular matrix or bound to cell wall components [40,43,45,46]. Similar effects have been reported for other plant by-products. Mushtaq [43] showed that a mixture of pectinase, protease, and cellulase improved the recovery of bioactive compounds from pomegranate peel, while Arnous and Meyer [47] demonstrated that pectinolytic enzymes enhanced polyphenol extraction from grape skins through the degradation of cell wall polysaccharides, promoting the release and transformation of flavonoids and phenolic acids. Comparable findings have also been reported for citrus by-products. Ruviaro et al. [36] demonstrated that enzyme mixtures were more effective than individual enzymes in promoting the transformation of phenolic compounds and the conversion of glycosylated flavonoids into their aglycone forms. Similarly, Van Hung et al. [37] observed increased levels of total phenolics, flavonoids, naringin, and hesperidin in pomelo peel extracts following enzymatic treatment. Wen [38] further showed that pectinase and Viscozyme L significantly enhanced the recovery of phenolic compounds, particularly flavonoids and their aglycones, from sweet orange peel through enzyme-assisted disruption of the cell wall structure. Overall, these studies support the present findings and indicate that enzymatic pretreatment prior to hydrodistillation has potential to improve the recovery of phenolic compounds from citrus peels, although its effectiveness depends on the citrus matrix, enzyme mixture composition, and extraction medium. The phenolic composition of each aqueous extract obtained from orange, mandarin, and clementine peels is described in detail in the following sections.

For orange peel, the observed mass fractions of individual phenolic compounds varied among pretreatments (Table 1). The combined enzymatic pretreatment in citrate buffer (HDB-REPCX) resulted in the highest sum of the mass fraction of quantified phenolic compounds (10.88 µg/g), although the overall variation in the total sum among pretreatments was relatively limited. Narirutin reached its highest observed mass fraction (0.59 µg/g) following xylanase pretreatment in purified water (HDW-REX) and the combined enzymatic pretreatment in citrate buffer (HDB-REPCX), while the combined enzymatic pretreatment in water (HDW-REPCX) resulted in a mass fraction of 0.51 µg/g, compared with 0.40 and 0.32 µg/g in the corresponding non-enzymatic controls (HDW-RE and HDB-RE, respectively). Quercetin (2.62 µg/g) and kaempferol (7.46 µg/g), the dominant flavonoids, were only moderately affected by the combined enzymatic pretreatment, with relatively small differences in their mass fractions among several pretreatment conditions. A comparable phenolic profile of orange peel extracts has been reported previously, with total phenolic contents reaching up to 5 mg/g [35], quercetin and kaempferol identified as the predominant flavonoids [41], and quercetin concentrations of up to 73 µg/g [28] and 420 µg/g [26].

Quercetin and kaempferol were also the dominant phenolic compounds in all analyzed aqueous extracts of mandarin peel. Higher mass fractions of these compounds were observed following some enzymatic pretreatments, particularly those performed in citrate buffer. The highest flavonoid contents were obtained following pretreatment with xylanase in buffer (HDB-REX) and the combined enzyme system in buffer (HDB-REPCX). Xylanase pretreatment (HDB-REX) produced the highest quercetin content (7.10 µg/g), as well as the highest kaempferol content (7.58 µg/g). Similarly, the combined enzymatic pretreatment in buffer (HDB-REPCX) yielded a kaempferol content of 7.50 µg/g. The combined enzyme treatment also markedly increased naringenin content, reaching 1.24 µg/g in purified water (HDW-REPCX) and 2.46 µg/g in citrate buffer (HDB-REPCX), compared with 0.27 and 0.23 µg/g, respectively, in the corresponding control samples (HDW-RE and HDB-RE, respectively). These mass fraction patterns suggest that the combined enzyme treatment promoted the release of flavonoids from the mandarin peel matrix. Although kaempferol was detected at relatively high levels in all samples, its highest concentrations were consistently observed following xylanase (HDB-REX) and enzyme mixtures (HDB-REPCX) in citrate buffer pretreatments. The concentrations of quercetin and kaempferol obtained in the present study agree with previous reports, although substantially higher values have been described when organic solvents were used for extraction, reflecting their greater efficiency in recovering flavonoids. For example, kaempferol contents of 16.85 µg/g [25] and 203.5 µg/g [32] have been reported, whereas quercetin reached 29.78 µg/g [25]. Likewise, Hayat et al. [32] reported a considerably higher naringenin concentration of 29.3 µg/g. Other identified phenolic compounds, including rutin, narirutin, quercetin-3-glucoside, kaempferol-3-glucoside, and luteolin, were present at lower mass fractions, which varied among pretreatments. Notably, quercetin-3-glucoside, kaempferol-3-glucoside, and luteolin were detected predominantly in enzyme-treated mandarin peels, suggesting an enhanced release of bound phenolics from the plant matrix. The highest sum of the mass fraction of quantified phenolic compounds was obtained in the HDB-REPCX aqueous extract (19.73 µg/g), representing an approximately 49% increase compared with the control sample (13.22 µg/g). Thus, among the conditions evaluated, the combined enzymatic pretreatment (REPCX) in citrate buffer was associated with the highest observed total mass fraction of quantified phenolic compounds in mandarin peel.

The most pronounced differences in the observed phenolic mass fractions among pretreatment conditions were found for clementine peel (Table 3). Xylanase pretreatment in purified water (HDW-REX) was associated with the highest mass fractions of several identified phenolic compounds. In particular, HDW-REX produced the highest mass fractions of quercetin (3.11 µg/g), kaempferol (10.70 µg/g), isorhamnetin (3.19 µg/g), naringenin (4.64 µg/g), and caffeic acid (13.25 µg/g). Consequently, the highest sum of the mass fraction of quantified phenolic compounds was also recorded for HDW-REX (34.94 µg/g), representing more than a threefold increase compared with the control sample (9.89 µg/g; HD). These observations indicate that, among the conditions evaluated, xylanase pretreatment in an aqueous medium was associated with the highest recovery of quantified phenolic compounds from clementine peel. Since no published studies have reported the phenolic composition of aqueous clementine peel extracts obtained under comparable conditions, direct comparison of individual phenolic compounds is not possible. Nevertheless, as clementine belongs to the *Citrus* genus, the predominance of quercetin and kaempferol observed in the present study is consistent with the review by M’Hiri et al. [41], who identified these flavonoids as the major phenolic constituents of citrus peels.

Although higher phenolic yields are generally reported for extraction methods employing organic solvents and advanced techniques, such as ultrasound- and microwave-assisted extraction, the present study indicates the potential of enzymatic pretreatment to enhance the recovery of phenolic compounds from aqueous hydrodistillation by-products without the use of organic solvents. This approach therefore may represent an environmentally friendly strategy for the valorization of citrus processing residues and may support the integration of hydrodistillation by-products into green extraction technologies. To the best of our knowledge, this is the first study to evaluate the effect of enzymatic pretreatment prior to hydrodistillation on the phenolic composition of aqueous hydrodistillation by-products (aqueous extract) obtained from orange, mandarin, and clementine peels. The findings indicate that these aqueous extracts, which are typically considered secondary by-products of essential oil production, represent a potential source of valuable phenolic compounds and could be further exploited within an integrated and sustainable citrus biorefinery approach.

## 3. Materials and Methods

### 3.1. Chemicals

The following chemicals were used: citric acid (Gram-Mol, Zagreb, Croatia), sodium hydroxide (Lach-Ner, Brno, Czech Republic), cellulase (from *Aspergillus niger*) (Sigma-Aldrich, Tokyo, Japan), pectinase (from *Aspergillus niger*) (Sigma-Aldrich, Buchs, Switzerland), xylanase (from *Thermomyces* sp., expressed in *Aspergillus oryzae*) (Sigma-Aldrich, Søborg, Denmark), and C9–C25 alkanes (Eurisotop, Saint-Aubin, France). Analytical standards of quercetin-3-glucoside (≥95%), kaempferol-3-*O*-rutinoside (≥95%), and naringenin (≥95%) were purchased from Sigma-Aldrich (Merck, St. Louis, MO, USA). Statistical analyses were performed using R software (version 4.5.3; R Foundation for Statistical Computing, Vienna, Austria).

### 3.2. Plant Material

Fresh citrus fruits used in this study were purchased from a local supermarket in Zagreb, Croatia. Specifically, fresh fruits of sweet orange (*Citrus* × *sinensis*), cv. Navel; mandarin (*Citrus reticulata*), cv. Unshiu; and clementine (*Citrus* × *aurantium*), cv. Clemenules, were obtained for the analyses.

### 3.3. Extraction Procedure

The procedure for the enzymatic pretreatment of citrus peels, as well as Clevenger hydrodistillation, has been described in detail in a previously published study [40]. Following hydrodistillation, essential oil, hydrolate, and an aqueous residue were obtained. Briefly, the enzymatic pretreatment included: (i) reflux extraction with enzymes (pectinase, REP; cellulase, REC; xylanase, REX; pectinase/cellulase/xylanase, REPCX) in purified water or citrate buffer (pH 5), as well as control pretreatments: (ii) reflux extraction without enzymes (RE) in purified water (HDW) or citrate buffer (pH 5) (HDB), which served as control samples. A control determination without prior pretreatment (HD) was also performed.

The activities of the enzymes (cellulase, pectinase, and xylanase) in purified water and citrate buffer (pH 5) were confirmed using the colorimetric 3,5-dinitrosalicylic acid (DNSA) method, as previously described [40]. Active hydrolysis was observed in purified water (pectinase 50.4 U/mL, cellulase 12.7 U/mL, xylanase 22.3 U/mL) and in citrate buffer (pectinase 41.5 U/mL, cellulase 25.3 U/mL, xylanase 13.5 U/mL).

The essential oil and hydrolate were separated one hour after the completion of Clevenger hydrodistillation, after the apparatus had cooled. The remaining aqueous residue, containing residual citrus peel material, was filtered through a funnel lined with filter paper to remove the peel residues. The resulting aqueous extract was stored at −18 °C until UPLC–MS/MS analysis.

### 3.4. UPLC–MS/MS Analysis of Phenolic Compounds

Phenolic compounds were analyzed using an ultra-performance liquid chromatography–tandem mass spectrometry (UPLC–MS/MS) system consisting of an Agilent 1290 RRLC system coupled to a triple quadrupole mass spectrometer (Agilent 6430, Santa Clara, CA, USA) equipped with an electrospray ionization (ESI) source operating in both positive and negative ionization modes (*m*/*z* 100–1000). The analytical procedure was performed according to Elez Garofulić et al. [48]. Chromatographic separation was achieved on a Zorbax Eclipse Plus C18 column (100 × 2.1 mm, 1.8 µm; Agilent Technologies, Santa Clara, CA, USA) maintained at 35 °C. The injection volume was 5 µL, and the flow rate was set at 0.3 mL/min. The total run time was 23 min. The mobile phase consisted of (A) water with 0.1% formic acid (*v*/*v*) and (B) methanol with 0.1% formic acid (*v*/*v*). Gradient elution was applied as follows: 0–1 min, 5% B; 1–10 min, 5–40% B; 10–15 min, 40–70% B; 15–18 min, 70–95% B; followed by a 5 min re-equilibration at initial conditions (5% B) prior to the next injection. Mass spectrometric conditions were optimized as follows: capillary voltage 4000 V in positive ion mode and 3500 V in negative ion mode; drying gas temperature 300 °C; drying gas flow rate 11 L/min; and nebulizer pressure 40 psi. High-purity nitrogen was used as both nebulizing and collision gas. Data acquisition and processing were performed using Agilent MassHunter Workstation software (version B.04.01). Phenolic compounds were identified by comparing retention times, precursor ions, and MS/MS fragmentation patterns with those of authentic standards, including quercetin-3-glucoside, kaempferol-3-*O*-rutinoside, apigenin, naringenin, and caffeic acid. Tentative identification of additional compounds was performed by comparison of MS/MS fragmentation data with previously reported literature values, as well as with available spectral databases. Quantification was performed using external calibration curves constructed with authentic standards. Due to the unavailability of some reference standards, selected compounds were quantified as follows: rutin, quercetin, and isorhamnetin were expressed as quercetin-3-glucoside equivalents; kaempferol and kaempferol-3-O-glucoside were quantified as kaempferol-3-*O*-rutinoside equivalents; narirutin was expressed as naringenin equivalents; and luteolin was quantified as apigenin equivalents. All calibration curves demonstrated good linearity within the working range (R^2^ > 0.99). Limits of detection (LODs) and limits of quantification (LOQs) were determined based on signal-to-noise ratios (S/N) of 3 and 10, respectively, under the optimized analytical conditions.

### 3.5. Statistical Analysis

All statistical analyses were performed using R software (version 4.5.3; R Core Team, Vienna, Austria) [49]. Data are presented as mean values of three repeated analytical measurements performed on the same prepared sample for each treatment condition (*n* = 3). These repeated measurements represent analytical rather than independent experimental replicates and were used to assess the repeatability of the analytical determination. Prior to statistical testing, the normality of residuals was assessed using the Shapiro–Wilk test, while homogeneity of variances was evaluated using Levene’s test. One-way analysis of variance, followed by Tukey’s honestly significant difference (HSD) post hoc test when applicable (*p* < 0.05), was retained as a descriptive statistical comparison of the repeated analytical measurements among pretreatment conditions. These statistical comparisons were not interpreted as evidence of treatment effects based on independent experimental replication. Accordingly, comparisons among treatments in the Results and Discussion were based primarily on the descriptive evaluation of the observed concentrations and trends. For visualization purposes, phenolic composition data were log-transformed using log(1 + x) to reduce the impact of highly abundant compounds (e.g., kaempferol and quercetin) and to improve the visualization of low-abundance metabolites. Heatmaps were generated using R-based visualization packages. Compounds that were not detected were assigned a value of zero for visualization purposes, as their concentrations were below the detection limit under the applied UPLC-MS/MS conditions. These values were therefore interpreted as below the detection limit rather than as confirmed absence of the corresponding compounds.

## 4. Conclusions

This study demonstrated that aqueous extracts obtained as by-products of citrus peel hydrodistillation contain phenolic compounds, with quercetin and kaempferol being predominant across the investigated citrus species. Differences in phenolic mass fractions were observed following enzymatic pretreatment, with the magnitude of these changes depending on the citrus species and extraction conditions. The highest sum of the mass fractions of quantified phenolic compounds was observed with the combined enzyme treatment in citrate buffer for mandarin peel and with xylanase pretreatment in water for clementine peel, while the response of orange peel was less pronounced and did not result in an increase in the sum of the mass fractions of quantified phenolic compounds. Overall, the results suggest a species-dependent response to enzymatic pretreatment, with the most pronounced increases in the observed mass fractions of phenolic compounds found for mandarin and clementine peels. These findings support the potential valorization of citrus peel hydrodistillation by-products within sustainable citrus processing.

## Figures and Tables

**Table 1:** Phenolic composition in orange (*Citrus sinensis*) peel water extracts determined by UPLC–MS/MS analysis.

*w*/(µg/g)	HD	HDW-RE	HDW-REP	HDW-REC	HDW-REX	HDW-REPCX	HDB-RE	HDB-REP	HDB-REC	HDB-REX	HDB-REPCX
Rutin ^a^	0.30 a	0.06 e	0.10 d	0.17 b	0.13 c	0.13 c	0.10 d	0.11 cd	0.10 d	0.23 ab	0.16 bc
Narirutin ^b^	0.45 cd	0.40 d	0.47 c	0.52 b	0.59 a	0.51 b	0.32 e	0.51 b	0.37 de	0.50 b	0.59 a
Isorhamnetin ^a^	0.14 a	0.13 a	0.15 a	0.12 a	0.15 a	0.15 a	0.06 b	0.05 b	0.05 b	0.06 b	0.05 b
Quercetin ^a^	2.48 bc	2.52 b	2.78 a	2.65 ab	2.50 bc	2.59 b	2.46 c	2.42 c	2.60 b	2.50 bc	2.62 ab
Kaempferol ^c^	7.18 b	7.36 ab	7.11 b	7.10 b	6.92 c	7.12 b	7.30 ab	7.42 ab	7.36 ab	7.41 ab	7.46 a
Total sum	10.54 b	10.48 bc	10.61 b	10.55 b	10.29 cd	10.50 bc	10.24 d	10.51 bc	10.48 bc	10.70 ab	10.88 a

Effect of different hydrodistillation pretreatments on the mass fraction of orange water extracts. HD—hydrodistillation without pretreatment (no-pretreatment control); HDW—hydrodistillation with water (no—enzyme control); HDB—hydrodistillation with buffer (no—enzyme control); RE—reflux extraction without enzyme; REP—reflux extraction with pretreatment assisted with enzyme pectinase; REC—reflux extraction with pretreatment assisted with enzyme cellulase; REX—reflux extraction with pretreatment assisted with enzyme xylanase; REPCX—reflux extraction with pretreatment assisted with mixture of enzymes (pectinase/cellulase/xylanase). Values are presented as means of three repeated analytical measurements of the same prepared sample (*n* = 3). Different lowercase letters (a–e) indicate differences among treatment means obtained from the repeated analytical measurements, according to one-way ANOVA followed by Tukey’s HSD test (*p* < 0.05). Since the replicates represent repeated analytical measurements rather than independent experimental replicates, these comparisons are descriptive and should not be interpreted as evidence of statistically significant treatment effects. ^a^ Quantified as quercetin-3-glucoside equivalents. ^b^ Quantified as naringenin equivalents. ^c^ Quantified as kaempferol-3-*O*-rutinoside equivalents.

**Table 2:** Phenolic composition in mandarin (*Citrus reticulata*) peel water extracts determined by UPLC–MS/MS analysis.

*w*/(µg/g)	HD	HDW-RE	HDW-REP	HDW-REC	HDW-REX	HDW-REPCX	HDB-RE	HDB-REP	HDB-REC	HDB-REX	HDB-REPCX
Rutin ^a^	0.17 e	0.46 bc	0.44 c	0.09 f	0.14 e	0.17 e	0.39 d	0.47 bc	0.63 ab	0.54 b	0.71 a
Kaempferol-3-*O*-rutinoside *	0.09 d	0.15 bc	0.14 bc	0.11 cd	0.11 cd	0.10 d	0.13 c	0.19 ab	0.12 c	0.11 cd	0.19 a
Narirutin ^b^	0.83 d	0.97 bc	0.90 cd	0.87 cd	1.05 ab	1.09 a	0.75 e	1.02 ab	0.81 de	0.47 f	0.80 de
Quercetin-3-glucoside *	0.00 f	0.12 e	0.08 e	0.00 f	0.09 e	0.05 e	0.46 d	0.44 d	0.60 c	1.74 a	0.65 b
Kaempferol-3-glucoside ^c^	0.00 e	0.00 e	0.00 e	0.00 e	0.00 e	0.10 d	0.20 c	0.00 e	0.14 cd	0.25 b	0.09 d
Isorhamnetin ^a^	0.10 b	0.08 c	0.14 a	0.11 ab	0.11 ab	0.11 ab	0.00 d	0.00 d	0.00 d	0.00 d	0.00 d
Quercetin ^a^	4.61 b	2.48 e	2.50 e	2.41 e	2.16 f	2.43 e	2.45 e	3.81 c	2.78 d	3.02 cd	7.10 a
Kaempferol ^c^	7.15 bc	7.24 abc	6.71 d	7.07 c	7.17 bc	7.18 bc	7.37 ab	6.84 cd	6.75 d	7.58 a	7.50 a
Luteolin *	0.00 c	0.00 c	0.00 c	0.00 c	0.00 c	0.00 c	0.00 c	0.21 ab	0.18 b	0.15 b	0.24 a
Naringenin *	0.27 d	0.27 d	0.12 e	0.26 d	0.26 d	1.24 c	0.23 d	1.97 b	1.43 c	0.32 d	2.46 a
Total sum	13.22 d	11.77 g	11.03 h	10.91 h	11.09 h	12.47 f	11.97 fg	14.95 b	13.43 d	14.17 c	19.73 a

Effect of different hydrodistillation pretreatments on the mass fraction of mandarin water extracts. HD—hydrodistillation without pretreatment (no-pretreatment control); HDW—hydrodistillation with water (no—enzyme control); HDB—hydrodistillation with buffer (no—enzyme control); RE—reflux extraction without enzyme; REP—reflux extraction with pretreatment assisted with enzyme pectinase; REC—reflux extraction with pretreatment assisted with enzyme cellulase; REX—reflux extraction with pretreatment assisted with enzyme xylanase; REPCX—reflux extraction with pretreatment assisted with mixture of enzymes (pectinase/cellulase/xylanase). Values are presented as means of three repeated analytical measurements of the same prepared sample (*n* = 3). Different lowercase letters (a–h) indicate differences among treatment means obtained from the repeated analytical measurements, according to one-way ANOVA followed by Tukey’s HSD test (*p* < 0.05). Since the replicates represent repeated analytical measurements rather than independent experimental replicates, these comparisons are descriptive and should not be interpreted as evidence of statistically significant treatment effects. ^a^ Quantified as quercetin-3-glucoside equivalents. ^b^ Quantified as naringenin equivalents. ^c^ Quantified as kaempferol-3-*O*-rutinoside equivalents. * Authentic standard.

**Table 3:** Phenolic composition in clementine (*Citrus clementine*) peel water extracts determined by UPLC–MS/MS analysis.

*w*/(µg/g)	HD	HDW-RE	HDW-REP	HDW-REC	HDW-REX	HDW-REPCX	HDB-RE	HDB-REP	HDB-REC	HDB-REX	HDB-REPCX
Rutin ^a^	0.11 bc	0.07 d	0.06 de	0.06 de	0.05 e	0.00 f	0.15 b	0.11 bc	0.00 f	0.21 a	0.00 f
Isorhamnetin ^a^	0.10 b	0.07 c	0.08 bc	0.06 c	3.19 a	0.05 c	0.19 b	0.00 d	0.05 c	0.00 d	0.06 c
Quercetin ^a^	2.55 bc	2.49 c	2.40 d	2.51 bc	3.11 a	2.61 b	2.55 bc	2.61 b	2.63 b	2.60 b	2.75 b
Kaempferol ^b^	7.13 d	7.33 d	7.35 d	7.44 d	10.70 a	8.09 bc	8.23 b	8.17 bc	7.79 cd	8.01 bc	7.84 cd
Naringenin *	0.00 d	0.00 d	0.00 d	0.00 d	4.64 a	0.43 c	0.23 c	0.00 d	0.00 d	0.60 b	0.00 d
Caffeic acid *	0.00 e	0.00 e	0.00 e	0.00 e	13.25 a	7.39 b	3.79 cd	2.28 d	4.03 c	3.39 cd	2.52 d
Total sum	9.89 d	9.96 d	9.89 d	10.06 d	34.94 a	18.58 b	15.14 c	13.16 e	14.50 cd	14.81 c	13.18 e

Effect of different hydrodistillation pretreatments on the mass fraction of clementine water extracts. HD—hydrodistillation without pretreatment (no-pretreatment control); HDW—hydrodistillation with water (no—enzyme control); HDB—hydrodistillation with buffer (no—enzyme control); RE—reflux extraction without enzyme; REP—reflux extraction with pretreatment assisted with enzyme pectinase; REC—reflux extraction with pretreatment assisted with enzyme cellulase; REX—reflux extraction with pretreatment assisted with enzyme xylanase; REPCX—reflux extraction with pretreatment assisted with mixture of enzymes (pectinase/cellulase/xylanase). Values are presented as means of three repeated analytical measurements of the same prepared sample (*n* = 3). Different lowercase letters (a–f) indicate differences among treatment means obtained from the repeated analytical measurements, according to one-way ANOVA followed by Tukey’s HSD test (*p* < 0.05). Since the replicates represent repeated analytical measurements rather than independent experimental replicates, these comparisons are descriptive and should not be interpreted as evidence of statistically significant treatment effects. ^a^ Quantified as quercetin-3-glucoside equivalents. ^b^ Quantified as kaempferol-3-*O*-rutinoside equivalents. * Authentic standard.

## Data Availability

The original contributions presented in this study are included in the article. Further inquiries can be directed to the corresponding authors.

## Supplementary Materials

The following supporting information can be downloaded at: https://www.mdpi.com/article/10.3390/plants15182871/s1, Figure S1. UPLC–MS/MS chromatograms of phenolic compounds in citrus peel aqueous extracts: (a) orange, (b) mandarin, and (c) clementine. Similar chromatographic profiles were observed across the different pretreatments. HD—hydrodistillation without pretreatment (no-pretreatment control); HDW—hydrodistillation with water (no-enzyme control); HDB—hydrodistillation with buffer (no-enzyme control); RE—reflux extraction without enzyme; REP—reflux extraction with pectinase-assisted pretreatment; REC—reflux extraction with cellulase-assisted pretreatment; REX—reflux extraction with xylanase-assisted pretreatment; REPCX—reflux extraction with combined enzyme pretreatment (pectinase/cellulase/xylanase). Orange peel extract: 1—Isorhamnetin; 2—Narirutin; 3—Rutin; 4—Quercetin; 5—Kaempferol. Mandarin peel extract: 1—Kaempferol-3-rutinoside; 2—Quercetin-3-glucoside; 3—Isorhamnetin; 4—Kaempferol-3-glucoside; 5—Naringenin; 6—Narirutin; 7—Rutin; 8—Quercetin; 9—Kaempferol. Clementine peel extract: 1—Caffeic acid; 2—Isorhamnetin; 3—Naringenin; 4—Quercetin; 5—Kaempferol.

## Abbreviations

The following abbreviations are used in this manuscript:

HD	hydrodistillation without pretreatment (no-pretreatment control)
HDW–RE	hydrodistillation with reflux extraction pretreatment in water (no-enzyme control)
HDW–REP	hydrodistillation with reflux extraction pretreatment assisted by pectinase enzymes in purified water
HDW–REC	hydrodistillation with reflux extraction pretreatment assisted by cellulase enzymes in purified water
HDW–REX	hydrodistillation with reflux extraction pretreatment assisted by xylanase enzymes in purified water
HDW–REPCX	hydrodistillation with reflux extraction pretreatment assisted by pectinase/cellulase/xylanase enzymes in purified water
HDB–RE	hydrodistillation with reflux extraction pretreatment in citrate buffer (no-enzyme control)
HDB–REP	hydrodistillation with reflux extraction pretreatment assisted by pectinase enzymes in citrate buffer
HDB–REC	hydrodistillation with reflux extraction pretreatment assisted by cellulase enzymes in citrate buffer
HDB–REX	hydrodistillation with reflux extraction pretreatment assisted by xylanase enzymes in citrate buffer
K-3-R	kaempferol-3-*O*-rutinoside
Q-3-Glc	quercetin-3-*O*-glucoside
K-3-Glc	kaempferol-3-*O*-glucoside
