# Supplementary material for: Phenolic Characterisation of Citrus Peel Aqueous Extract: By-Products of Hydrodistillation and the Effect of Enzymatic Pretreatment
Source: Plants (Basel). 2026 Sep 19;15(18):2871. doi: 10.3390/plants15182871 (PMC13611369; doi:10.3390/plants15182871)
Supplement: Supplementary file 1 [file plants-15-02871-s001.zip › plants-4532121-supplementary.pdf]

plants

Supplementary Material

Phenolic Characterisation of *Citrus* Peel Aqueous Extract: By-Products of Hydrodistillation and the Effect of Enzymatic Pretreatment

Antonela Ninčević Grassino *, Marija Penić, Sandra Pedisić, Sven Karlović and Maja Dent *

Faculty of Food Technology and Biotechnology, University of Zagreb, Pierottijeva 6, 10000 Zagreb, Croatia; doktoricamare@gmail.com (M.P.); spedisic@pbf.hr (S.P.); skarlovi@pbf.hr (S.K.)

* Correspondence: aninc@pbf.hr (A.N.G.); maja.dent@pbf.unizg.hr (M.D.); Tel.: +385-1-4605-062 (A.N.G.); +385-1-4605-096 (M.D.)

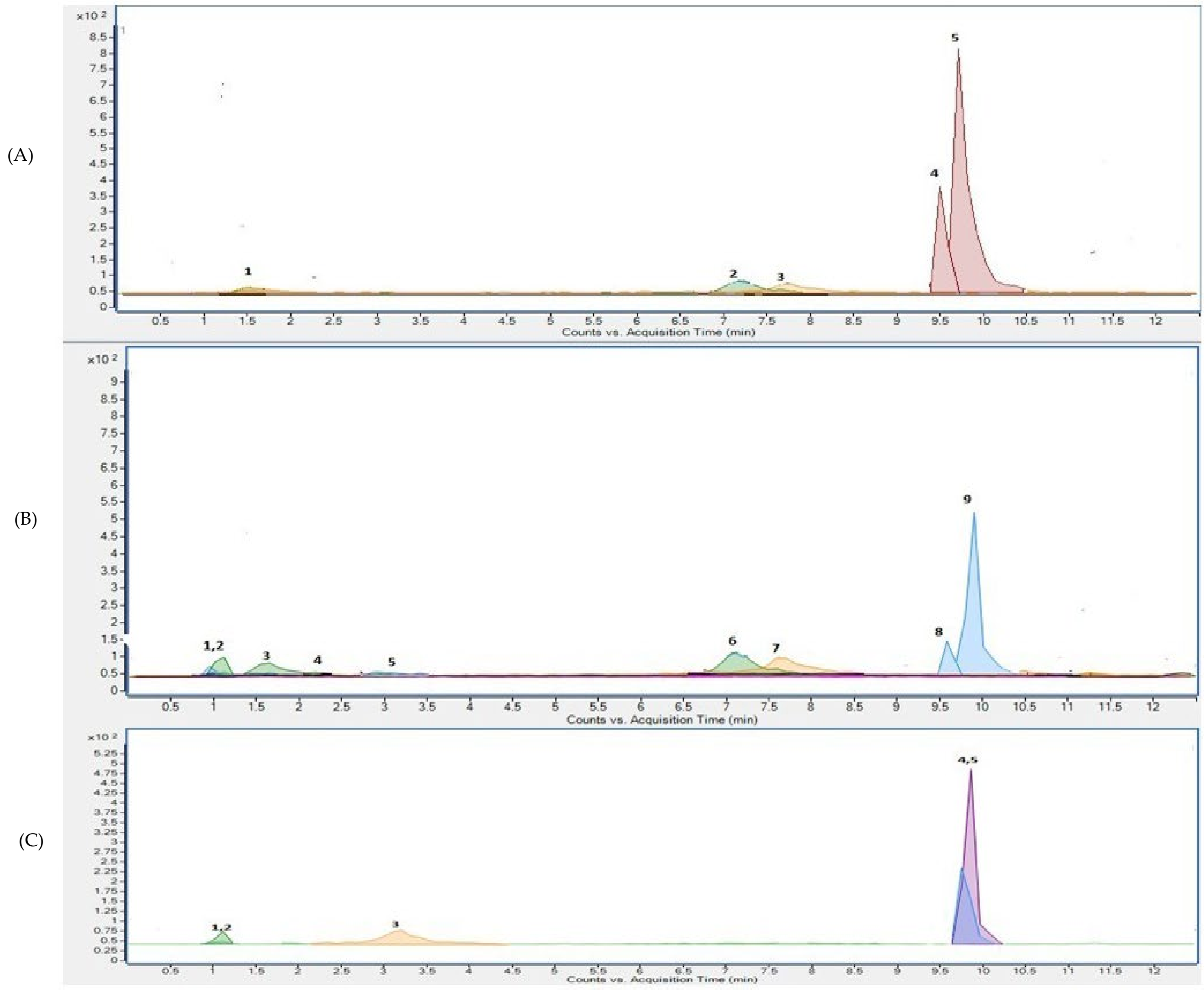

Figure S1. UPLC–MS/MS chromatograms of phenolic compounds in citrus peel aqueous extracts: (a) orange, (b) mandarin, and (c) clementine. Similar chromatographic profiles were observed across the different pretreatments. HD—hydrodistillation without pretreatment (no-pretreatment control); HDW—hydrodistillation with water (no-enzyme control); HDB—hydrodistillation with buffer (no-enzyme control); RE—reflux extraction without enzyme; REP—reflux extraction with pectinase-assisted pretreatment; REC—reflux extraction with cellulase-assisted pretreatment; REX—reflux extraction with xylanase-assisted pretreatment; REPCX—reflux extraction with combined enzyme pretreatment (pectinase/cellulase/xylanase). Orange peel extract: 1—Isorhamnetin; 2—Narirutin; 3—Rutin; 4—Quercetin; 5—Kaempferol. Mandarin peel extract: 1—Kaempferol-3-rutinoside; 2—Quercetin-3-glucoside; 3—Isorhamnetin; 4—Kaempferol-3-glucoside; 5—Naringenin; 6—Narirutin; 7—Rutin; 8—Quercetin; 9—Kaempferol. Clementine peel extract: 1—Caffeic acid; 2—Isorhamnetin; 3—Naringenin; 4—Quercetin; 5—Kaempferol.
